# Knowledge, practices and expectations of preventive care: a qualitative study of patients attending government general outpatient clinics in Hong Kong

**DOI:** 10.1186/s12875-018-0740-7

**Published:** 2018-05-09

**Authors:** Denise Y. S. Tam, Yvonne Y. C. Lo, Wendy Tsui

**Affiliations:** 1 Department of Family Medicine and Primary Healthcare, Hong Kong West Cluster, Hospital Authority, North Wing, Room 601, 6/F, Tsan Yuk Hospital, 30 Hospital Road, Hong Kong, Hong Kong; 2 Wellness Family Medical Practice, Room 605, Hang Seng Tsim Sha Tsui Building, 18 Carnarvon Road, Hong Kong, Hong Kong

**Keywords:** Primary health care, Prevention, Patients’ perspective

## Abstract

**Background:**

Evidence-based preventive care recommendations have been well established, but studies have persistently reported gaps between these recommendations and general practitioners’ practices in providing preventive care. Many studies have explored factors that affect the delivery of preventive care from the perspectives of the practitioners, but relatively few have evaluated the patients’ point of view. The purpose of this study was to explore patients’ understanding of preventive care, the actions they were taking in terms of preventive health and their expectations from family doctors in providing preventive care.

**Methods:**

A qualitative study was conducted based on one-on-one in-depth interviews. Twenty-eight patients without chronic illnesses were purposively recruited from government general outpatient clinics in Hong Kong. The interviews took place between November 2013 and February 2014.

**Results:**

The participants’ knowledge of preventive care was limited, and their preventive practices were mostly restricted to healthy lifestyle practices. They rarely obtained individualised preventive care advice from doctors. Screening investigations were initiated after symptoms had already occurred, and the decision of what to check was arbitrary. Few of the participants knew what they wanted from their doctors in terms of preventive care.

**Conclusions:**

These findings show significant gaps between evidence-based preventive recommendations and patients’ current knowledge and practice, and show the need for a wider spectrum of preventive care education and reliable sources to provide individualised and affordable preventive assessment and screening services. Most importantly, primary care providers must take a more proactive role to provide preventive services.

## Background

Preventive care is an important component of family medicine. Family doctors are in a unique position to assess, educate and counsel individuals to improve their general health status, facilitate screening and prevent the onset of disease [1]. However, overseas studies have persistently reported gaps between evidence-based recommendations and general practitioners’ practices in providing preventive care [2, 3]. The 2004 Commonwealth Fund International Health Policy Survey examined the experiences of thousands of adults in Australia, Canada, New Zealand, the United Kingdom and the United States and found an overall lack of emphasis on prevention in primary care practice [3].

Similar shortcomings were noted in a study conducted in government general outpatient clinics in Hong Kong [4]. Overall, few health promotion activities were offered, and significantly more lifestyle counselling was geared for patients with chronic diseases in whom the undesirable damage from risky behaviour had already occurred. Similar deficiencies were noted in the private health care sector. A local audit of the practice of anticipatory care in a private outpatient clinic showed that even after interventions, most preventive categories, including immunisation, cancer screening and elderly functional assessment, failed to meet the target standards [5].

Hong Kong has no structured population-based health screening program. Some patients schedule check-ups via commercially available packages that offer screening investigations. Why do patients choose such services? Is it for preventive purposes? How do they choose where to go and for what to screen? Assessment of patients’ attitudes and current behaviour in terms of screening is important to understand their preventive care needs.

Various studies have explored factors that affect the delivery of preventive care. Many were conducted from the perspectives and experiences of the practitioners [6–10]. In contrast, studies that directly evaluate patients’ perspectives are comparatively limited [11–15].

In 2012, the Primary Care Office released a reference framework for preventive care for adults in the primary care setting in Hong Kong [16]. This framework provided evidence-based recommendations in areas like vaccination, practice of healthy lifestyles, dental health, chronic illness screening, cancer screening, functional disability assessment, mental disorder screening, polypharmacy and adverse drug reaction screening and social support assessment. However, despite the availability of this comprehensive framework, little is known about local patients’ views and beliefs regarding preventive care, their current health-seeking behaviour regarding prevention and their expectations of preventive care from their primary care doctors. In family medicine, in which a collaborative care model is emphasised, it is crucial to understand patients’ knowledge, current practices and expectations to facilitate delivery of quality preventive care.

The objectives of this study were:

- To examine the participants’ understanding about preventive care;
- To explore participants’ current preventive practices;
- To examine participants’ attitudes regarding commercial screening packages, their reasons for choosing particular services and the extent to which such screening was carried out; and
- To elucidate participants’ expectations of primary care doctors in providing preventive services.

## Methods

This study was approved by the Institutional Review Board of The University of Hong Kong/Hospital Authority Hong Kong West Cluster. All subjects gave informed consent to participate.

### Study design

We chose a qualitative method for this study because little was known about the study topic. Also, an exploratory approach was useful for evaluation of perceptions and expectations. Through this approach, we wished to capture the participants’ understanding, contexts and reasons associated with their preventive care experiences and expectations. Qualitative study can be done in the form of individual interviews or focus-group interviews. We believed that one-on-one interviews were a better option for our study because they would likely touch upon sensitive issues such as personal medical history and financial status.

### Sample and data collection

The study sample was drawn from two government general outpatient clinics in Hong Kong. Patients without chronic diseases were included because we were especially interested in primary prevention and secondary prevention in terms of screening. The following inclusion and exclusion criteria were applied. Patients were recruited if they were older than 18 years of age and were willing to participate in the study. Patients were excluded if they had any chronic illness or a hearing problem; were unable to communicate in Cantonese; were mentally incapacitated, rendering participation in the study inappropriate (e.g., mental retardation or dementia); or refused to participate in the study.

As a qualitative study, the sample size was not pre-determined. Participants were purposively sampled to ensure, as far as possible, an even representation of the various sex and age categories (18 to 29 years, 30 to 39 years, 40 to 49 years, 50 to 59 years and 60 years and above).

We gave potential participants an information sheet that explained the aims of the study. All participants were asked to sign a written consent form before enrolment. A $50 (Hong Kong dollars) supermarket coupon was issued to each participant as an incentive to take part in the study.

We interviewed all participants individually in a quiet room to ensure confidentiality. Basic demographic data were collected, and the interviews were semi-structured and guided by pre-set questions (Table 1). The questions were developed based on the study objectives and were pilot tested on two subjects to ensure the clarity and understandability of each question. The lead investigator (DT) conducted all interviews between November 2013 and February 2014. All interviews were audiotaped and transcribed verbatim. Data collection was continued until thematic saturation was reached, as agreed by the first and second coders after review of the raw data.

**Table 1:** Interview guide

1. What is preventive care? Would you give some examples? If the respondent could not answer at all, give hints: Smoking cessation and regular exercise are both examples of effective preventive measures, can you think of others? 2. Have you intentionally done something to improve your own health or to screen for disease? If yes: what have you done? If no: Why not? 3. Where did you get the health information concerning preventive care? Aside from the mentioned sources, where else would do you prefer to obtain such information? 4. Have you ever had physical check-up? If yes: Under what circumstance was it done? How did you choose where to go for the check-up? How did you choose what to do for the check-up? What did the check-up include? Was a doctor available to explain the result? If no: Why not? If you were to have physical check-up, how would you choose what to do? Where to go? 5. How do you think of commercially available check-up packages? 6. Do you have a doctor that you regularly attend? Have you ever discussed with this doctor anything in terms of preventive care? If yes, what have you discussed before? If no, why not? 7. If given the opportunity, what would you want to discuss with your doctor in terms of preventive care?	

### Analysis

The data were analysed by a content analysis approach in a stepwise manner. The qualitative research program N Vivo 10 was used to enhance the accuracy of the coding process. The lead investigator (DT) carried out the coding. First, the audiotapes were reviewed, and the transcripts were read. The content of each transcript was then coded from key words and phrases. Similar codes were grouped into categories. New categories were added and adjusted as appropriate for an ongoing, non-linear analysis, and themes were generated. To avoid bias in the coding process, the second investigator (YL) independently coded a sample of the transcripts. The two coders discussed and compared their findings at different stages to ensure consensus in coding and theme identification. There were no disagreements in coding for which a consensus could not be reached. Representative quotations were selected to exemplify the findings.

## Results

### Participant characteristics

We interviewed 28 participants, whose characteristics are shown in Table 2. We purposefully selected participants to provide equal representations of various age groups and sexes. Most participants had secondary or higher education. We included participants with and without medical insurance coverage and with and without a family doctor.

**Table 2:** Participant Characteristics

Marital status	Household income	Education	Health insurance	Has a family doctor?
Single	4	Tertiary	No	No
Married	6	Secondary	Yes	Yes
Married	4	Secondary	Yes	No
Married	5	Secondary	Yes	Yes
Widowed	6	Secondary	No	No
Married	6	Secondary	Yes	No
Single	2	Secondary	No	No
Single	4	Tertiary	No	No
Married	2	Secondary	Yes	No
Married	2	Primary	Previously	Yes
Single	3	Tertiary	Yes	Don’t know
Married	2	Secondary	Previously	No
Single	2	Tertiary	No	No
Single	3	Tertiary	Yes	Yes
Single	5	Tertiary	Yes	No
Single	3	Secondary	Yes	No
Married	5	Tertiary	Yes	No
Widowed	1	Primary	Yes	No
Single	5	Tertiary	Yes	Yes
Single	2	Tertiary	No	No
Married	1	Secondary	Yes	Yes
Married	5	Tertiary	Yes	No
Single	3	Tertiary	Yes	No
Married	4	Secondary	Yes	Yes
Divorced	3	Secondary	No	Yes
Single	2	Tertiary	Yes	No
Married	1	Secondary	Yes	No
Married	5	Tertiary	No	No

Key**:** Monthly income (Hong Kong Dollars): 6: Refused to answer; 5: ≥$40,000; 4: $30,000- < $40,000; 3: $20,000- < $30,000; 2: $10,000- < $20,000; 1: <$10,000

### Understanding of preventive care

Only a quarter of the participants could accurately define preventive care as taking actions to maintain health before the onset of disease. Four participants believed that to take action shortly after symptoms occurred also counted as preventive measures. The remaining participants could not tell specifically the purpose of preventive care. When probed they were only able to give examples of practices that they believed would keep one healthy. One participant mentioned that preventive care was needed when one was old but did not elaborate on the reasons that elderly people need preventive practices. Diet control (20 participants), exercise (15 participants) and hygienic measures (11 participants) were the most commonly mentioned preventive methods. Other methods included screening tests (7 participants), maintaining good mental health (6 participants), getting sufficient rest (6 participants), taking supplements or health products (5 participants), avoiding smoking or drinking (4 participants) and keeping warm (4 participants). Some participants mentioned specific evidence-based methods such as receiving vaccinations (3 participants) and checking cervical smears (2 participants).

The participants’ preventive health concepts were mixed with ideas from traditional Chinese medicine and alternative medicine. For example, whilst participants understood that getting enough rest might enhance their health, one participant chose to sleep before a certain time in the evening to allow the body to remove ‘toxins’ effectively, according to Chinese Medicine theory:

*‘To sleep early can enhance your body’s efficiency in removing toxins. For example, our liver starts to remove toxins after 11 o’clock at night.’* (Subject 11)

The emphasis on the intake of Chinese herbal soups and proprietary Chinese medicine are also examples of cultural influence on health beliefs:

*‘For preventive care, our family makes herbal soup once in a while.’* (Subject 17)

*‘I think prevention is when we use proprietary Chinese medicine to protect our liver and kidneys… I am referring to those over-the-counter medications that we can buy at Mannings or Watsons (local pharmacy chains). I think this is what preventive care is about.’* (Subject 20)

The participants’ concepts of what it means to eat well might also be influenced by alternative medicine. For example, one participant mentioned that one should choose less ‘acidic’ fluid for drinking:

*‘Our family knows that mineral water is better than distilled water. This is because mineral water is less acidic. I think women should limit their intake of acidic fluid.’* (Subject 14)

### Sources of preventive information

The participants’ knowledge of preventive care comes from a variety of sources. More than half of the participants obtained preventive information from the television or the Internet. Television sources included television programs, news and government-funded advertisements. Internet sources included websites or Internet forums. Only two participants specified the use of government health-related websites. Other information channels included health pamphlets distributed from hospitals and clinics and health talks held at elderly centres, community centres, schools and libraries. In addition, about one third of the participants relied upon their friends and/or family as sources of preventive information. One patient expressed that he would depend on his own instincts. Only one participant referred to a health care professional for preventive advice.

Almost all participants believed that the availability of preventive care information was adequate. However, some of them recognised a lack of individualised advice:

*‘Everyone’s condition is different. Some preventive measures may apply to certain individuals only, but not to me…’* (Subject 15)

At the same time, some participants were uncertain about the accuracy of the available information:

*‘The information delivered through television or newspaper is nothing new, but sometimes I wonder if all the information delivered is true.’* (Subject 17)

### Current behaviour and attitudes regarding screening

Most participants had negative feelings towards commercial screening packages and took a rather passive role in initiating such check-ups. They generally lacked confidence in these packages, regarding them as overpriced, profit-driven, excessive and unreliable. One patient also expressed the concern that screening in itself might produce false reassurances and thus perpetuate one’s unhealthy lifestyle.

Amongst our participants, primary care doctors had little role in initiating screening tests; only one participant completed a check-up as suggested by his doctor. Whilst some participants had initiated screening tests with the primary aim of understanding their own body condition and to prevent the onset of illnesses, about the same number of participants sought check-ups because symptoms already existed or because of past illnesses. When participants were feeling well, they generally did not see the need for screening. This perception was universal across both genders and different age groups.

*‘I know I may go for [a physical examination], but I do not have much concern because there is no sign to show that anything is going wrong in my body; therefore, I did not go.’* (Subject 5)

*‘The first time I went was because I had discomfort.’* (Subject 10)

*‘I did not perceive the urgency. When I feel well, I would not do anything.’* (Subject 19)

When they went for screening, the participants had a vague idea of what should be checked. In general, they believed that older people warranted more thorough check-ups and that older people should be screened for chronic illnesses like hypertension, diabetes and heart diseases. Apart from that, the participants’ beliefs about testing needs were quite arbitrary and were often related to their self-perceived health risks, which might or might not be based on medical grounds.

*‘I would like to see if there is a chance of getting cancer or to check for HIV or hepatitis B. (Why do you want to check for such things?) Because those are more significant diseases.’* (Subject 7)

*‘(What would you screen for when you go for a health check?) I think there is a greater urgency to examine the colon because it is an urban disease…more young people and increasing numbers of people are getting colon diseases, I think this is why examining the colon is a more popular option. Other [physical examinations] may examine the bones and the eyes, so far I don’t think I have needs in these areas…other check-ups may examine the lungs, I think I am ok in this area…mainly, I would like to screen the parts that I worry about the most.’* (Subject 2)

Cost was another significant influence on behaviour related to preventive care. Regardless of their level of perceived health risks, participants might be reluctant to participate in screening because of the cost. The participants sometimes chose a particular health check package simply because it was the cheapest.

The participants had a superficial idea of what constitutes a quality screening service. Most looked for ‘reputable places’ or ‘big centres’ to ensure the quality of their health check; for ‘reputable places’, participants generally referred to places that were recommended by friends or relatives.

*‘(How do you choose where to go?) Ask my friends, because I have no clue, see where they think is good for check-up then I would choose that place.’* (Subject 10)

*‘Bigger centres are more reliable, more credible…because as a company they need to run their business; they would not want to ruin their own brand name.’* (Subject 16)

*‘…Depends on where others have visited, which place offered more comprehensive and detailed examination…and which place had better feedback…’* (Subject 19)

### Expectations on primary care doctors in terms of preventive advice

#### Experience

Few participants stated that their doctors had given them preventive advice.

#### Perceived barriers

When asked why they had not asked their doctors for preventive care advice, most participants expressed that their doctors were too busy. This perception was shared amongst participants of various sex and age groups.

*‘I felt that he was very busy, I would not talk about these things (preventive information) for no reason and then demand that my doctor reply to me.’* (Subject 3)

*‘There is no reason to talk about such things (preventive advice)…It would hinder the next patient from seeing the doctor. We should have empathy; the doctor was busy, and there was no reason for us to talk about these and that…this is meaningless.’* (Subject 6)

*‘There were only few minutes in the consultation, nothing more could be said.’* (Subject 9)

*‘My doctor had many patients lining up to see him, I had to wait 1 to 2 hours every time, I did not want to hinder his work.’* (Subject 14)

It was obvious that anticipatory care was considered ‘inappropriate’ in a busy clinic. Most participants perceived the presenting illness as the main agenda of the consultation. If they did not have symptoms, the patients would not consult a doctor solely for anticipatory advice.

*‘Everybody is like this…you consult a doctor when you are sick, you would not go to a doctor for prevention.’* (Subject 10)

*‘Seeing a doctor is mainly for treating your disease.’* (Subject 11)

A lack of initiation by the doctor was another barrier. Some participants expected their doctor to be the one who initiated preventive care in a consultation.

*‘It was over 20 years…I felt that if I have been with this doctor for so long and he knew my record, he should have told me if there was anything I needed to know or do about disease prevention…but he never said anything.’* (Subject 14)

A lack of patient awareness of the importance of preventive care was another barrier.

*‘…I was not aware that I should ask about prevention.’* (Subject 8)

*‘I don’t know how to ask people about these things, I felt I was being passive…I hope someone could tell me and then I would learn about it; otherwise, I would remain ignorant about it (preventive care).’* (Subject 12)

#### Expectations

When asked about what they would like to ask their primary care doctors concerning anticipatory care, more than one third of participants stated that they would like to obtain advice on current symptoms or past illnesses. One third of participants did not know what to ask or had nothing to ask. Less than one third of participants could raise specific questions related to prevention. Specifically, these participants were interested in learning about their current health status, determining whether they need a physical examination or where they should go to get one, and acquiring age-specific health advice and other dietary advice.

## Discussion

We found that the participants’ knowledge of preventive care was limited and that their preventive practices largely focused on healthy lifestyle practices. They rarely obtained first-hand or individualised preventive care advice from doctors, and they lacked the means to evaluate the heath information they received from other sources. Screening tests were initiated after symptoms had already occurred, and the decision of what to check was arbitrary. When given the opportunity, less than one third of the participants knew what they wanted to ask their doctors in terms of prevention. Perceived barriers to seeking preventive advice from doctors include an excessive focus on presenting illnesses, a lack of doctor initiative, a lack of awareness of the importance of prevention and time pressure.

Our participants’ preventive knowledge and behaviour focused largely on lifestyle practices, which is in line with the results of a local study that explored patients’ attitudes about health and self-care [17]. This local study found that patients’ self-care referred mostly to diet and exercise and included massage, Tai chi, herbal remedies, dietary supplements and vitamins and traditional ‘food therapies’ such as certain types of soups. Our participants also mentioned the use of Chinese herbal therapies, supplements and ‘keeping warm’ as preventive methods; such practices are in line with the context of our local Chinese culture. The lack of awareness and low uptake of evidence-based recommendations for adult vaccination and cancer screening were in line with the findings from the 2012 local census, which found that only 5.7% of Hong Kong adults had received a flu vaccination within the past year and that only 6.8% of women had undergone a cervical smear within the past 3 years [18]. Certain preventive care domains such as functional disability assessment were barely mentioned by the participants. Such findings reveal significant discrepancies between the health recommendations and actual practices. One explanation might be insufficient health literacy. There is consistent evidence that people’s level of health literacy is associated with their health behaviour [19]. A local study explored the relationship between health literacy and health behaviour in Chinese parents with preschool children regarding the prevention of seasonal influenza. It found that Hong Kong parents had inadequate knowledge and that their reported preventive behaviour did not concur with their actual health practices [20]. A study on the development of a Health Empowerment Programme to improve the health of working poor families in Hong Kong is currently underway, and health literacy is one of its intervention components [21]. This study may shed light on strategies that may improve one’s health practices and enhance disease prevention.

A Hong Kong survey of the public’s perception of primary health care and their expectations of related services reported that 75.5% of subjects either agreed or strongly agreed that primary care doctors should provide preventive care [22]. However, for various reasons, very few participants in our study received or sought anticipatory care from primary care doctors. First, compared to medical staff, patients tend to assign higher priority to acute or minor conditions than to preventive check-ups for chronic conditions [23]. This lack of patient concern might lead to doctors’ lack of initiative to provide anticipatory care. Existing studies of doctors’ points of view have also reported patient resistance as one of the most common barriers to the provision of health-promoting activities [6–8, 10].

Second, some patients did not actively seek anticipatory care during consultation because they did not know what to ask. Our findings show that, when given the opportunity, few participants could raise questions related to prevention. Patients may not know what preventive measures were relevant to them, which may prevent them from seeking preventive care from their family doctors [11]. This finding highlights the importance of family doctors in initiating anticipatory advice.

Third, time pressure was another barrier. Our participants generally believed that doctors were too busy to give anticipatory advice. The existing literature on doctors’ perceptions also reported a lack of time as a major barrier to the delivery of preventive care [6–8, 10]. In Hong Kong, the average duration of a consultation in a government clinic is about 6 to 7 min. One study in the United States suggested that simply providing the grade A preventive care recommendations of the US Task Force would require a primary care doctor 2 h per day in a typical practice of 2500 patients [24]. Previous studies had indicated that a shorter consultation time was associated with fewer preventive activities [25] and that a longer consultation time was associated with increased performance of preventive activities [26].

Fourth, we found that participants had a low level of perceived health risk in the absence of symptoms. They considered health as the absence of symptoms and commonly believed that healthy people did not need to consult a doctor [17]. The classic Health Belief Model proposed that although a person may perceive himself or herself as being susceptible to illness, action to reduce that risk will not be taken unless a cue to action is present [27]. Our findings suggest that asymptomatic patients generally do not perceive the need for prevention. Therefore, they could not be expected to take the lead in terms of preventive care.

Finally, we were worried to see that even if the subjects participated in health screening, they tended not to seek professional advice beforehand. It would be problematic if patients were left to their own discretions in terms of health screening, because their choices are often arbitrary and may not fit their specific medical needs. Also, a study on preventive services offered by private hospitals in Hong Kong showed that not all evidence-based preventive activities were being offered and that many unproven or even possibly harmful services were provided [28]. In our study, only one participant was concerned about false reassurance as a result of negative screening results; none of the others raise any issues about the possibility of harm from inappropriate screening. This finding again could be related to the participants’ insufficient level of health literacy.

This study is first of its kind to explore the views and behaviour of local patients regarding preventive care. The qualitative approach of this in-depth interview is appropriate, and the results derived are informative. We believe that many of the findings regarding the participants’ beliefs and attitudes might not be easily elicited with quantitative research methods.

Our study is not without limitations. The two government general outpatient clinics used for patient recruitment were not randomly selected, and both are located on Hong Kong Island, which is generally more affluent than other parts of Hong Kong. However, our sampling frame did include patients of both genders and various ages and socio-economic statuses. Patients who do not speak Cantonese were also under-represented. These patients comprise a small proportion of the Hong Kong population and may not have the same access to health care services as their Chinese-speaking counterparts. Because our participants were recruited from public general outpatient clinics, their views and attitudes may differ from those who only attend private doctors for their health problems. Nevertheless, the aim of this study, as with all other qualitative studies, was not to bring about findings to represent the general population, but to determine themes related to preventive care issues.

Our study has highlighted deficiencies that must be addressed to facilitate better delivery of preventive care amongst patients who attend public general outpatient clinics.

There is a need for a wider spectrum of preventive care education that is not limited to the promotion of a healthy lifestyle and that also includes evidence-based cancer screening, cardiovascular disease screening, functional assessment and other aspects as outlined in the Primary Care Office preventive care framework. At the time this manuscript was written, the Hong Kong government had launched an age-based colorectal cancer screening program. The effect of this program on patients’ preventive care awareness and practices is yet to be determined. There is also a need for reliable sources – governmental, non-governmental or private – to provide affordable and individualised assessment, preventive advice and screening services so that asymptomatic patients may become better aware of their potential health risks and take appropriate action. The relationship between preventive care and the level of health literacy in Hong Kong also requires further exploration. Primary care doctors should be more proactive in initiating anticipatory care, and their role in providing primary preventive care should be further promoted in the community. In view of limited consultation times, some possible solutions include the use of computer-generated prevention summaries and reminders [29] and the recruitment of practice nurses to provide preventive care assessment.

## Conclusions

Preventive care is an important component of family medicine. This study identified gaps between local evidence-based preventive recommendations and the participants’ current knowledge and practices. Our findings indicate a need for a wider spectrum of preventive care education and reliable sources to provide individualised and affordable preventive assessment, counselling and screening services. Most importantly, primary care doctors must take a more proactive role in advocating for and providing preventive care services.
